# Does More Sedentary Time Associate With Higher Risks for Sleep Disorder Among Adolescents? A Pooled Analysis

**DOI:** 10.3389/fped.2021.603177

**Published:** 2021-08-10

**Authors:** Yanjie Zhang, Sitong Chen, Chengyao Wang, Xiaoyi Zhang, Liye Zou, Xinli Chi, Can Jiao

**Affiliations:** ^1^Exercise Psychophysiology Laboratory, Institute of KEEP Collaborative Innovation, School of Psychology, Shenzhen University, Shenzhen, China; ^2^Physical Education Unit, School of Humanities and Social Science, The Chinese University of Hong Kong–Shenzhen, Shenzhen, China; ^3^Institute for Health and Sport, Victoria University, Melbourne, VIC, Australia; ^4^School of Psychology, Shenzhen University, Shenzhen, China

**Keywords:** adolescents, global school-based health survey, insomnia, anxiety, sedentary behavior

## Abstract

**Purpose:** To investigate the association between sedentary behavior and anxiety-induced sleep disorder at a global perspective.

**Methods:**A total of 254,924 adolescents (mean age: 14.45 ± 1.42 years; 52.8% girls) who participated in the Global School-Based Student Health Survey were included for analysis. Self-reported questionnaires assessed anxiety-induced sleep disorder and sedentary behavior. Multivariable logistic regression analysis and countrywide meta-analysis were used for investigating the association between sedentary behavior and anxiety-included sleep disorder.

**Results:**The results showed that sedentary time was linearly associated with higher OR of anxiety-related sleep disorder in adolescents across the countries and that 8 h or more per day increased the OR by 2.17 times. Countrywide meta-analysis showed that 8 h or more per day of sedentary behavior yielded an OR = 1.40 (95% CI = 1.34–1.46) of anxiety-induced sleep disorder. Moreover, the association between sedentary behavior and sleep anxiety was significant in adolescents over the age of 11 years regardless of sex.

**Conclusions:**The findings from this study suggest that as sedentary behavior increases, sleep disorders also increase, independently of sex among adolescents. Effective preventive strategies are needed to be taken to decrease sedentary behavior that could be used to improve mental health and sleep quality among adolescents.

## Introduction

Sufficient sleep in adolescents is extremely important for promoting physical and mental health development (1, 2). However, in modern society, sleep disorder is becoming a serious public health issue among adolescents accompanied by high prevalence (3). For example, statistical result from the 2014 *Health behavior in school-aged children* report showed that 36% of adolescents in England had sleep disorder (4). Available studies indicate that sleep disorder is associated with many mental disorders, especially including anxiety (5, 6), whereby a reciprocal relationship between sleep disorder and anxiety has been established, indicating that sleep disorder leads to an increase in anxiety problems and vice versa (6, 7). Both sleep disorder and anxiety not only contribute to a number of adverse health events or behavior, such as cognitive impairment, obesity, chronic disease, and abuse of electronic products among adolescents (8–10) but also result in a heavy economic burden for individuals or society (11).

At present, cognitive behavior therapy and pharmacological interventions are considered as the two main approaches that have been found to be effective in treating sleep disorder and anxiety (12, 13). Based on previous studies, although cognitive behavior therapy produces considerable efficacy in treatment of sleep disorder, it may be too static and boring for adolescents to comply with the therapy (14). Likewise, pharmacological interventions are liable to cause adverse effects for adolescents in the clinic (13). Considering the issues encountered in the actual treatment and in order to prevent or treat psychological health problems, it is critical to identify cost-effective and adolescent-friendly interventions, as well as understanding the potential risk factors related to anxiety-related sleep disorder.

A growing body of studies have documented that healthy lifestyle (e.g., physical activity, healthy eating and no smoking) is a potentially modifiable factor; in particular, physical activity is easy to carry out and can positively improve people's sleep patterns, such as total sleep time, sleep onset latency, and sleep efficiency (15, 16). On the contrary, sedentary behavior is prevalent among teenagers in daily life (17). Sedentary behavior is defined as “any waking behavior characterized by an energy expenditure ≤ 1.5 metabolic equivalents (METs), while in a sitting or reclining posture,” such as screen time, driving, reading, and study time (18). Since most adolescents engage in a lot of sedentary activities, it greatly increases their risk of poor health outcomes (e.g., sleep disorder, mental disorder, and cardiovascular disease) (19, 20). For example, Costigan et al. conducted a systematic review and found that there was a positive relationship between screen-based sedentary behavior and depression among adolescent girls (21). A cross-sectional study conducted in China showed that youths who spent more time on sedentary behavior were more likely to report poor sleep quality and anxiety (22).

So far, previous studies failed to offer a global perspective on the association between sedentary behavior and anxiety-related sleep disorder among adolescents. Some studies investigated this association based on a single country or parts of the countries around the world. For example, a cross-sectional study focused on low- and middle-income countries, showing that sedentary behavior was associated with anxiety-related sleep disorder among 12–15-year adolescents (23). Another study supported this association based on Brazil representative samples (24). Although there is a relationship between sedentary behavior and anxiety-induced sleep disorder in some countries, this relationship based on global countries, different age, and sex remains to be investigated (25).

To have an entire understanding of the aforementioned relationship, the current study included data from 77 countries based on the Global School-based Health Survey (GSHS) to investigate the association between sedentary behavior and anxiety-related sleep disorder among adolescents aged 11–17 years old globally.

## Methods

### Survey Data

The publicly available data in the present study was retrieved from the GSHS (http://www.who.int/chp/gshs and http://www.cdc.gov/gshs). The aim of this research project was detailed in previous study (26). The GSHS content was extracted from the Youth Risk Behavior Survey (YRBS), with satisfactory reliability (27). The survey with multiple-choice response options was translated into the local language to record the students' responses in each country. When selecting the participants, a cluster sample design, which is both standardized and scientific based on two stages, was adopted so that the survey could be carried out in each country. During the first stage, when selecting the schools, the probability remained proportional to the sampling size. During the second stage, classrooms including students aged 11–17 years within each selected school were randomly selected. All the students were instructed to fill out the questionnaire regardless of their gender or age. Data collection was conducted during a normal classroom session. The GSHS research protocol was approved before starting the survey by each national government administration or ethics committee. Each student (or parents and school officials) signed an informed consent before starting the survey. During the survey period, anonymous and voluntary participation was used to protect student privacy. Data were weighted for non-response and probability selection. All recent datasets of each country and the variables consisting of sedentary time and anxiety-related sleep problem were used for analysis. A total of 77 countries (data from the 2003 to 2019 survey) were surveyed in this study. Supplementary Table S1 summarizes the characteristics of respondents in different countries in detail.

### Anxiety-Related Sleep Disorder

Anxiety-related sleep disorder had been described by using a question, “During the last year, how often did you have insomnia because of anxious feelings?” The possible answers consisted of “always,” “most of the time,” “sometimes,” “rarely,” and “never.” Furthermore, if “always” and “most of the time” were selected, then the respondent was regarded as having a sleep disorder associated with anxious feelings according to the previous study (23).

### Sedentary Behavior (Exposure Variable)

Sedentary behavior was described based on the answers to the question of “how long did you spend on chatting with friends, playing computer games, watching television or other activity done when you sit?”, and the possible answers consisted of <1, 1–2, 3–4, 5–6, 7–8, and >8 h/day. It is worth mentioning that the time spent on class, homework, or sleep had been excluded from the sedentary time. In the present study, the dichotomous variable had been adopted for analysis, that is, 3 h per day or not (23). The question was derived from the National Health and Nutrition Examination Survey (NHANES) questionnaire, which was widely used in previous studies (17, 23).

### Control Variables

The control variables were age, sex, physical activity, and food insecurity. Because the socioeconomic status as a variable had been excluded from the GSHS survey, food security served as a substitute of socioeconomic status, and this was consistent with a previous study (27). Food insecurity was measured using a question, “During the past month, how often were you hungry because there was not enough food in your home?,” and the possible answers consisted of “most of the time/always,” “rarely/sometimes,” and “never” (28). Physical activity was assessed based on the answers to the question “how many days did you spend at least 60 min on physical activity during the last week?” Participants were regarded as having sufficient physical activities if they answered 5–7 days with more than 60 min of physical activities in the past 7 days, in accordance with previous studies (27, 29).

### Statistical Analysis

SPSS 22.0 and R studio 14.1 were used for statistical analysis. For descriptive analyses, the prevalence of sedentary behavior and anxiety-related sleep disorder was calculated using the SPSS. Then, multivariable logistic analyses were conducted to investigate the association between sedentary behavior and anxiety-related sleep disorder. Based on the classification of a previous study, we categorized sedentary behavior into <3 h per day and ≥3 h per day (30). The country-wise and sex-wise multivariable logistic regression analyses were not adjusted for country and sex, respectively. All other regression analyses had been adjusted based on age, sex, physical activity, and food insecurity. Additionally, because there was significant interaction between age and sex, stratified analysis was performed for age and sex. An integrated estimate was calculated through conducting a meta-analysis with fixed effects model and 95% confidence interval (CI) in R Studio based on the results of country-wise analyses. Higgin's *I*^2^ was used to assess the level of between-country heterogeneity. Here, *I*^2^ values were classified as low (25%), moderate (50%), and high (75%) level of heterogeneity (31). In order to obtain country-representative estimates, sampling weight and cluster sampling design of the surveys were used for analyses. Results from the logistic regression analysis were calculated as odds ratios (ORs) with 95% CI. The *p* < 0.05 was set as the statistical significance level for all analyses.

## Results

**Figure 1:**
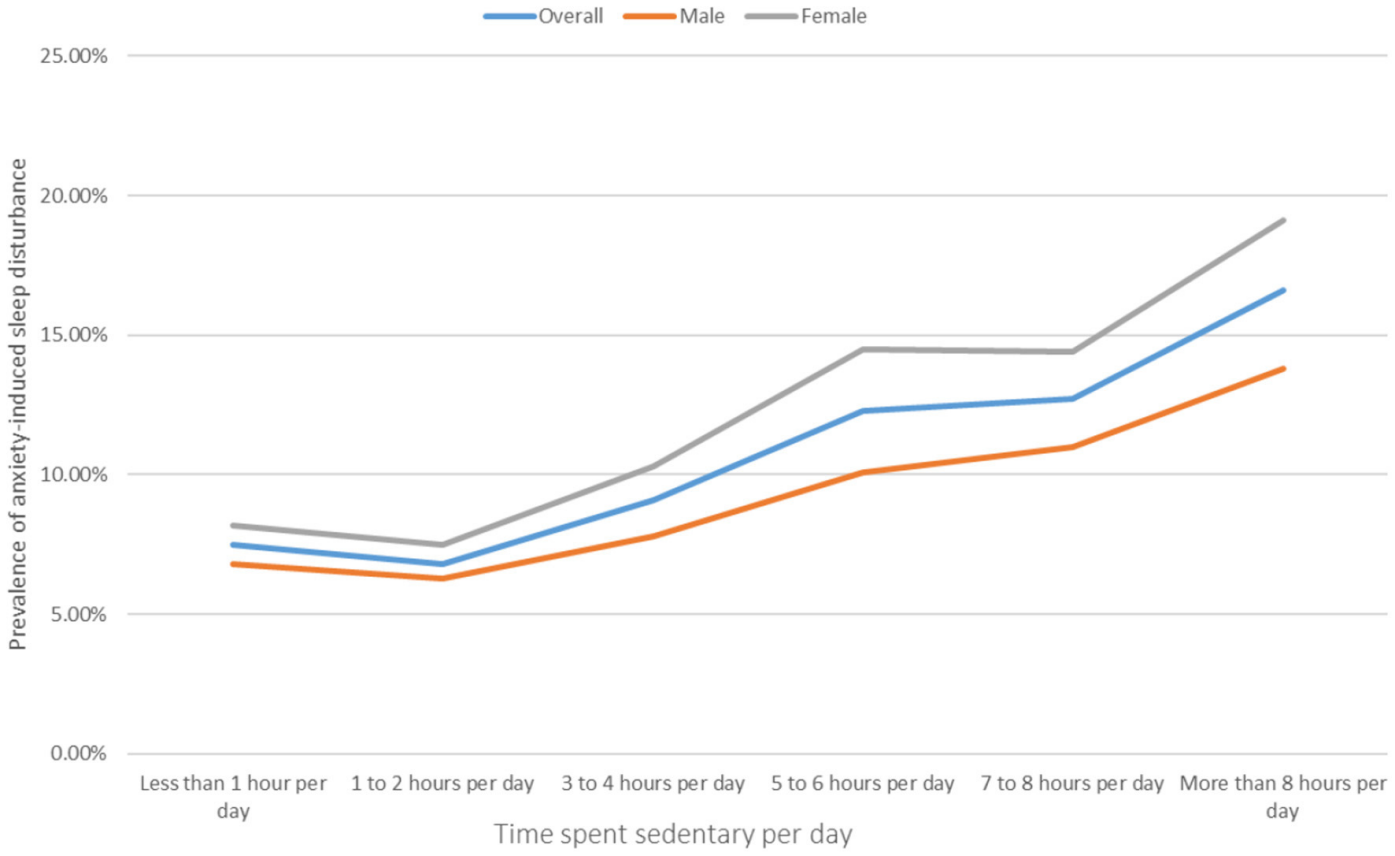
Prevalence of anxiety-induced sleep disturbance by the time spent sedentary. The time spent sedentary per day was defined as answering “ <1 h per day,” “1–2 h per day,” “3–4 h per day,” “5–6 h per day,” “7–8 h per day,” or “> 8 h per day” to the question “Time spent sitting on usual day?.” Anxiety-induced sleep disturbance was defined as answering “most of the time” or “always” to the question “During the past 12 months, how often have you been so worried about something that you could not sleep at night?”

A total of 254,924 adolescents aged 11–17 years (mean age: 14.45 ± 1.42; 52.8% girls) from 77 countries globally were included in this study. The overall prevalence of anxiety-related sleep disorder among adolescents was 8.3% (95% CI = 8.0–8.6%), and according to the categorizations of different sedentary behavior time, the prevalence of sedentary behavior was as follows: 38.3% (<1 h per day), 32.9% (1–2 h per day), 16.4% (3–4 h per day), 6.0% (5–6 h per day), 2.3% (7–8 h per day), and 4.2% (>8 h per day). The prevalence of sedentary behavior, which was more than 3 h per day, fluctuated from 10.6% (Nepal) to 65.6% (Barbados) while the prevalence of anxiety-related sleep disorder fluctuated from 3.5% (Venezuela) to 27.2% (Samoa) after age and sex adjustment (see Supplementary Table S1). The prevalence of anxiety-related sleep disorder increased linearly with the increase in time spent sedentary behavior; in particular, the sedentary time was more than 1–2 h per day. However, in the overall and gender-specific samples, the prevalence of anxiety-related sleep disorder at sedentary behavior of 1–2 h per day was slightly lower than that of <1 h per day (see Figure 1). Likewise, the risk of anxiety-related sleep disorder was significantly reduced by 9.0% in the odds for the sedentary behavior of 1–2 h per day compared with <1 h per day under multivariable logic regression. However, the increased odds for anxiety-related sleep disorder was associated with the time spent sedentary behavior (except for less than or equal to 2 h per day) in all samples (see Table 1). Overall, the >8 h per day of sedentary behavior resulted in a 2.17 (95% CI = 1.94–2.42) times greater odds for anxiety-related sleep disorder compared with <1 h per day. This result was also observed in both genders (see Table 1). The relationship between sedentary time and anxiety-related sleep disorder at different ages is shown in Table 2. This significant relationship was not observed in sedentary time <4 h per day, except for adolescents aged 13 years old with a sedentary time of 3–4 h/day (OR = 1.29, 95% CI = 1.05–1.59). On the contrary, this relationship was observed in sedentary time (5–6 h/day, 7–8 h/day, and >8 h/day) among adolescents aged 12–17 years old, except for adolescents aged 16 years old (5–6 h/day), 12 years old, and 17 years old (7–8 h/day) in Table 2.

**Table 1:** The relationship between sedentary time and anxiety-induced sleep disturbance estimated multivariable logistic regression (overall and by gender).

**Time spent sedentary**	***N***	**Overall^a^**		**Male^b^**		**Female^c^**	
		**OR**	**95% CI**	**OR**	**95% CI**	**OR**	**95% CI**
<1 h/day	81,539	ref		ref		ref	
1–2 h/day	79,681	0.90	[0.83, 0.98]	0.93	[0.82, 1.05]	0.88	[0.80, 0.98]
3–4 h/day	49,554	1.17	[1.07, 1.27]	1.16	[1.03, 1.31]	1.18	[1.06, 1.31]
5–6 h/day	20,205	1.65	[1.47, 1.85]	1.52	[1.27, 1.83]	1.77	[1.53, 2.05]
7–8 h/day	7,652	1.60	[1.37, 1.87]	1.54	[1.20, 1.96]	1.66	[1.34, 2.06]
>8 h/day	16,293	2.17	[1.94, 2.42]	1.98	[1.68, 2.33]	2.33	[2.02, 2.70]

*Anxiety-induced sleep disturbance was defined as answering “most of the time” or “always” to the question “During the past 12 months, how often have you been so worried about something that you could not sleep at night?” CI, confidence interval; OR, odds ratio; ref, Reference*.

a *Adjusted for country, gender, went hungry past 30 days, and physical activity*.

b, c *Adjusted for country, went hungry past 30 days, and physical activity*.

**Table 2:** The relationship between sedentary time and anxiety-induced sleep disturbance estimated on multivariable logistic regression at different ages.

**Age**	***N***	** <1 h/day**	**1–2 h/day**	**3–4 h/day**	**5–6 h/day**	**7–8 h/day**	**>8 h/day**
		**OR, 95% CI**	**OR, 95% CI**	**OR, 95% CI**	**OR, 95% CI**	**OR, 95% CI**	**OR, 95% CI**
11	2,843	ref	1.35 [0.68, 2.68]	1.73 [0.76, 3.94]	2.58 [0.71, 9.30]	2.50 [0.54, 11.56]	2.21 [0.83, 4.96]
12	17,427	ref	0.98 [0.78, 1.22]	1.30 [0.98, 1.74]	2.74 [1.76, 4.25]	1.11 [0.56, 2.22]	3.60 [2.19, 5.91]
13	49,735	ref	0.84 [0.70, 1.02]	1.29 [1.05, 1.59]	1.75 [1.35, 2.27]	1.84 [1.14, 2.97]	2.30 [1.79, 2.94]
14	61,669	ref	0.89 [0.76, 1.03]	1.13 [0.96, 1.32]	1.79 [1.40, 2.29]	1.41 [1.04, 1.89]	2.36 [1.95, 2.87]
15	58,315	ref	0.97 [0.84, 1.12]	1.11 [0.94, 1.32]	1.59 [1.29, 1.97]	1.50 [1.11, 2.02]	1.91 [1.55, 2.36]
16	44,262	ref	0.76 [0.62, 0.93]	1.00 [0.83, 1.21]	1.20 [0.93, 1.56]	1.65 [1.20, 2.28]	1.75 [1.36, 2.25]
17	20,673	ref	1.08 [0.87, 1.35]	1.26 [0.98, 1.61]	1.60 [1.10, 2.32]	1.56 [0.98, 2.48]	2.17 [1.58, 2.98]

*Anxiety-induced sleep disturbance was defined as answering “most of the time” or “always” to the question “During the past 12 months, how often have you been so worried about something that you could not sleep at night?” CI, confidence interval; OR, odds ratio; ref, Reference*.

*Adjusted for country, gender, went hungry past 30 days, physical activity*.

**Figure 2:**
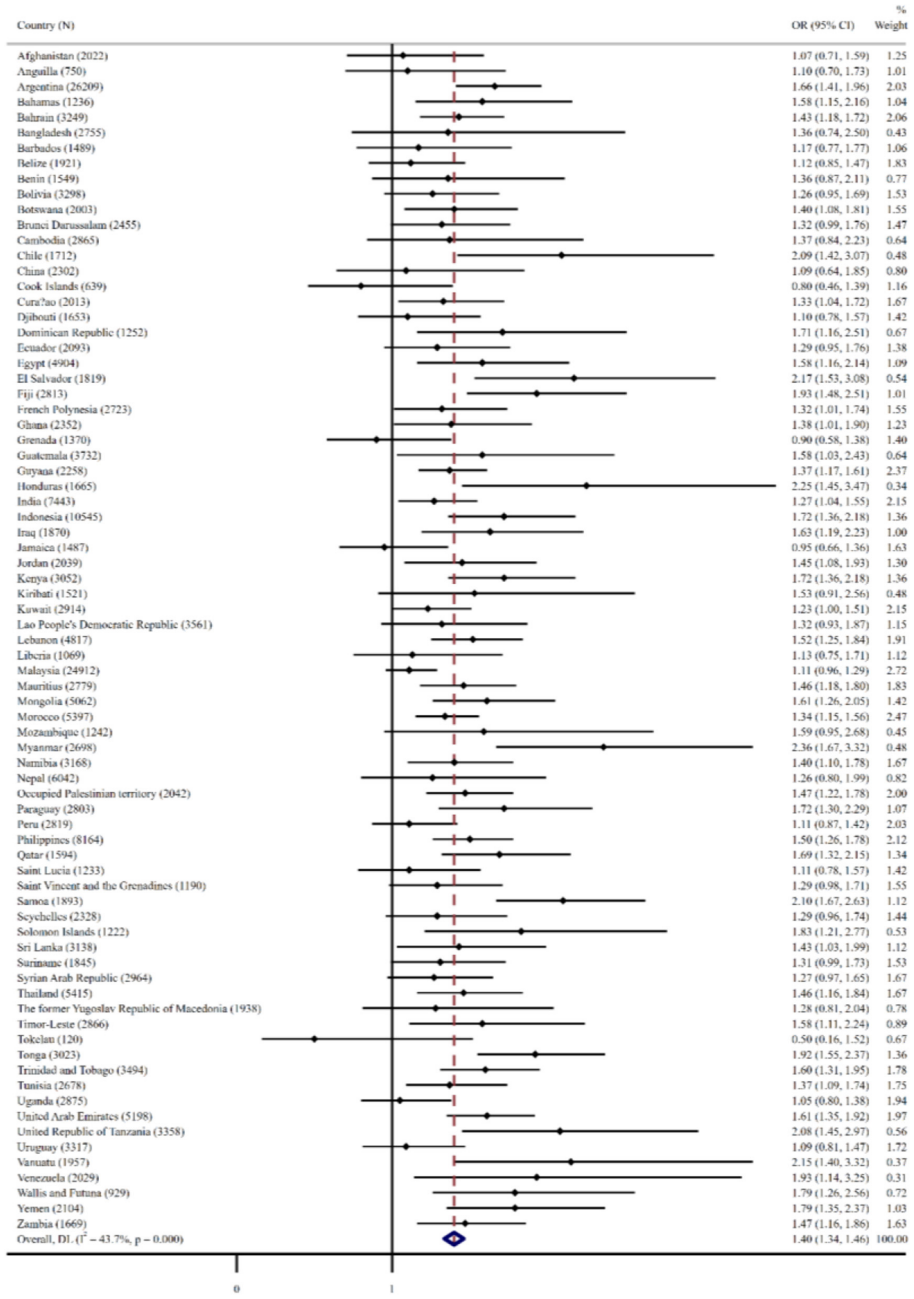
The relationship between anxiety-induced sleep disturbance and more than 3 h of the time spent sedentary behavior in different countries by multivariable logistic regression. Anxiety-induced sleep disturbance was defined as answering “most of the time” or “always” to the question “During the past 12 months, how often have you been so worried about something that you could not sleep at night?” The regression models are adjusted age, gender, went hungry past 30 days, and physical activity. The overall result was estimated by fixed effect model. OR, odds ratio; CI, confidence interval; N, sample size.

Based on the cutoff point of 3 h or more per day, Figure 2 depicts the relationships between anxiety-related sleep disorder and ≥3 h per day of sedentary behavior in different countries. The significant association between the greater odds of anxiety-related sleep disorder and the sedentary behavior for a long time was observed in 46 of 77 countries. The meta-analysis with the fixed effect model produced a whole estimation with OR = 1.40 (95% CI = 1.34–1.46) and a between-country heterogeneity of *I*^2^ = 43.7%. Furthermore, there was a significant association between sedentary behavior and anxiety-related sleep disorder in overall adolescents. Age and sex stratification analyses showed a significant association, except for 11-year-old boys and girls (see Table 3). In addition, the prevalence of anxiety-related sleep disorder at sedentary time (≥3 h/day) decreased linearly with age compared to sedentary time (<3 h/day). Both boys and girls had similar trends (see Table 3). The overall prevalence of sedentary behavior (≥3 h/day) increases with age from 23.4% at 11 years old to 38.1% at 17 years old. The similar trends were observed in both boys (22.5–35.8%) and girls (22.5–40.5%) (see Supplementary Table A1).

**Table 3:** The relationship between sedentary time (≥3 h/day) and anxiety-induced sleep disturbance estimated multivariable logistic regression based on age (overall and by gender).

**Age**	***N***	**Overall^a^**			**Boy^b^**			**Girl^c^**		
		**OR**	**95% CI**	**Prevalence of anxiety-induced sleep (%)^d^**	**OR**	**95% CI**	**Prevalence of anxiety-induced sleep (%)^d^**	**OR**	**95% CI**	**Prevalence of anxiety-induced sleep (%)^d^**
11	2,843	1.77	[1.06, 2.94]	7.8%	1.61	[0.77, 3.36]	8.3%	1.92	[0.81, 4.52]	7.5%
12	17,427	1.80	[1.43, 2.27]	6.3%	1.61	[1.16, 2.24]	6.7%	2.15	[1.65, 2.81]	6.1%
13	49,735	1.67	[1.46, 1.91]	6.9%	1.64	[1.35, 2.00]	6.4%	1.63	[1.36, 1.95]	7.5%
14	61,669	1.54	[1.38, 1.70]	7.6%	1.49	[1.26, 1.77]	6.7%	1.57	[1.36, 1.82]	8.7%
15	58,315	1.37	[1.22, 1.53]	8.9%	1.16	[0.98, 1.38]	7.3%	1.55	[1.32, 1.82]	10.7%
16	44,262	1.38	[1.21, 1.57]	10.9%	1.34	[1.10, 1.62]	9.3%	1.47	[1.24, 1.75]	12.7%
17	20,673	1.43	[1.19, 1.71]	11.0%	1.38	[1.09, 1.75]	9.9%	1.49	[1.15, 1.91]	12.1%

*Anxiety-induced sleep disturbance was defined as answering “most of the time” or “always” to the question “During the past 12 months, how often have you been so worried about something that you could not sleep at night?” CI, confidence interval; OR, odds ratio*.

*The reference value of sedentary time is <3 h/day*.

a *Adjusted for country, gender, went hungry past 30 days, and physical activity*.

b, c *Adjusted for country, went hungry past 30 days, and physical activity*.

d *(%) The prevalence of anxiety-induced sleep disturbance at different ages*.

## Discussion

The current study aimed to investigate the association between sedentary behavior and anxiety-related sleep disorder among 254,924 adolescents aged 11–17 years from 77 countries globally. Understanding this association is helpful for adolescents to change their lifestyle to reduce anxiety-related sleep problems for improving mental health. The findings from this study showed that sedentary time was linearly associated with higher OR of anxiety-related sleep disorder in adolescents across the countries. The 8 h or more per day of sedentary behavior increased the OR by 2.17 (95% CI = 1.94–2.42)] times of anxiety-related sleep disorder compared with that of <1 h per day. The time in sedentary behavior seems to vary according to age; however, 8 h or more manifests itself as a risk for all adolescents. Additionally, the integrated estimation of all countries revealed that adolescents spending ≥3 h per day of sedentary behavior had 1.40 (95% CI = 1.34–1.46) times higher OR for anxiety-related sleep disorder compared to the <3 h per day of sedentary behavior. The association between sedentary behavior and anxiety-related sleep disorder was significant across all the subgroups of adolescents except for 11-year-old boys and girls. However, the OR of anxiety-related sleep disorder by ≥3 h per day of sedentary behavior decreased with age compared to the <3 h per day of sedentary behavior irrespective of sex.

From the perspective of a single country or low- and middle-income countries, previous studies have found a positive relationship between sedentary behavior and anxiety-related sleep disorder in adolescents aged 12–15 years (23). The findings of the present study from a global perspective confirmed this association and suggested that spending sedentary time more than 8 h per day was associated with higher OR of anxiety-related sleep disorder among adolescents aged 11–17 years globally, regardless of sex compared with the study by Vancampfort et al. (23). Meanwhile, our finding showed a higher OR of overall integrated estimation without heterogeneity among adolescents across multiple countries. The findings of the present study added to the evidence that there was a significant relationship between sedentary behavior and sleep anxiety among adolescents (both boys and girls) aged more than 11 years old, and that the overall ORs of anxiety-related sleep disorder by 3 h/day of sedentary time decreased with age among adolescents, and the relationship was similar in both boys and girls.

The possible explanation is that the older adolescents are more likely to be exposed to heavy academic tasks than younger adolescents and even spend more screen time on academic-related activities for increased academic performance. Academic performance has been linearly associated with self-esteem (32), and high self-esteem is an important factor linked to lower level of anxiety and sleep disorder (33, 34). Another explanation is that younger adolescents are more susceptible to screen use (e.g., TV, electronic games) compared to older adolescents (35). Thereby, heavier screen use can result in sleep disorder among adolescents (36). In addition, due to the limited number of covariates in the present study, other confounders (e.g., stress and health condition) may also contribute to influence the association between sedentary behavior and anxiety-related sleep disorder among adolescents. For example, stress has been recognized as an important factor in sleep disorder (37, 38). Previous review studies suggested that high sedentary times predicted a high risk of stress, which resulted in sleep disorder (39, 40). Therefore, future studies are warranted to examine the impact of these confounders on this association.

The findings of the present study should be considered with several limitations. First of all, all analysis data were based on a cross-sectional survey, and a causal relationship was not inferred. Further follow-up studies were warranted to examine the relationship among adolescents. Second, it did use the subjective measurement approach to report sedentary time instead of objective measurement (e.g., accelerometer), and the study time was excluded according to the report from the original survey. Thus, an accurate sedentary time was not obtained and might even appear to be underestimated. Likewise, only one question about anxiety-related sleep disorder was used to measure insomnia, but this question has been widely used to provide effective measurement data with research (23, 25). Third, sedentary types were not included in the study. Different types of sedentary behaviors may also be associated differently with anxiety-related sleep disorders. For example, talking with friends in a sitting posture can be different from the impact of watching TV on sleep. Fourth, due to the data extracted from an established database, the information of some confounders (e.g., health conditions, family economic status, parental education, and place of residence) cannot be obtained and explored.

Notably, the current study involves some strengths. Nationally representative data from multiple countries with large sample sizes of adolescents can improve the generalization of the results to prevent anxiety-related sleep disorder among adolescents. Meanwhile, effective interventions are expected for reducing sedentary behavior time and enhancing sleep quality in early adolescents, especially for those adolescents who have a sedentary time of more than 8 h per day.

## Conclusion

The present study suggests that increased sedentary behavior is associated with high prevalence of anxiety-related sleep disorder in adolescents globally. Similarly, this association is observed in both boys and girls. Further studies are needed to understand the cause-and-effect relationship between sedentary behavior and sleep disorder among adolescents and to design rigorous experiments in preventing sedentary behavior.

## Data Availability Statement

The datasets presented in this study can be found in online repositories. The names of the repository/repositories and accession number(s) can be found in the article/Supplementary Material.

## Ethics Statement

Ethical review and approval was not required for the study on human participants in accordance with the local legislation and institutional requirements. Written informed consent to participate in this study was provided by the participants' legal guardian/next of kin.

## Author Contributions

YZ, SC, LZ, and CJ: conceptualization. YZ, SC, CW, XZ, LZ, XC, and CJ: methodology and formal analysis. YZ, SC, CW, XZ, LZ, XC, and CJ: writing—draft manuscript, review, and editing. All authors contributed to the article and approved the submitted version.

## Conflict of Interest

The authors declare that the research was conducted in the absence of any commercial or financial relationships that could be construed as a potential conflict of interest.

## Publisher's Note

All claims expressed in this article are solely those of the authors and do not necessarily represent those of their affiliated organizations, or those of the publisher, the editors and the reviewers. Any product that may be evaluated in this article, or claim that may be made by its manufacturer, is not guaranteed or endorsed by the publisher.
