# Supplementary material for: Does More Sedentary Time Associate With Higher Risks for Sleep Disorder Among Adolescents? A Pooled Analysis
Source: Front Pediatr. 2021 Aug 10;9:603177. doi: 10.3389/fped.2021.603177 (PMC8382689; doi:10.3389/fped.2021.603177)
Supplement: Supplementary file 1 [file Data_Sheet_1.docx]

**Table S1.** The prevalence of anxiety-induced sleep disturbance and sedentary in different countries

| Country | Year | Response rate (%)^1^ | N | Sedentary behavior (%)^2,3^ | Anxiety-induced sleep disturbance (%)^4,5^ |
| --- | --- | --- | --- | --- | --- |
| Afghanistan | 2014 | 79 | 2022 | 24.5 (19.1-30.7) | 23.0 (18.9-27.7) |
| Anguilla | 2016 | 88 | 750 | 60.1 (54.9-65.1) | 9.9 (7.9-12.5) |
| Argentina | 2012 | 71 | 26209 | 50.1 (48.6-51.5) | 8.9 (8.1-9.7) |
| Bahamas | 2013 | 78 | 1236 | 54.8 (51.6-57.9) | 13.7 (11.5-16.2) |
| Bahrain | 2016 | 89 | 3249 | 59.6 (54.7-64.3) | 17.9 (14.9-21.2) |
| Bangladesh | 2014 | 91 | 2755 | 15.4 (12.1-19.3) | 4.1 (3.2-5.3) |
| Barbados | 2011 | 73 | 1489 | 65.6 (62.4-68.6) | 9.9 (8.3-11.7) |
| Belize | 2011 | 88 | 1921 | 37.6 (34.3-41) | 11.9 (10.3-13.8) |
| Benin | 2016 | 78 | 1549 | 25.0 (20.7-29.9) | 18.3 (14.8-22.3) |
| Bolivia | 2012 | 88 | 3298 | 24.4 (21.7-27.3) | 7.2 (6.4-8.0) |
| Botswana | 2005 | 95 | 2003 | 34.2 (31.7-36.8) | 19.6 (18.3-20.9) |
| Brunei Darussalam | 2014 | 65 | 2455 | 56.3 (53.5-59) | 10.3 (9-11.8) |
| Cambodia | 2013 | 85 | 2865 | 11.6 (9.4-14.4) | 5.0 (4.3-5.9) |
| Chile | 2005 | 85 | 1712 | 36.2 (32.8-39.7) | 8.2 (6.7-9.9) |
| China | 2003 | 99 | 2302 | 21.8 (19.1-24.8) | 4.6 (3.6-5.7) |
| Cook Islands | 2015 | 65 | 639 | 45.1 (41.5-48.8) | 13.8 (11.4-16.7) |
| Curaçao | 2015 | 96 | 2013 | 61.2 (58.5-63.8) | 10.8 (9.3-12.5) |
| Djibouti | 2007 | 83 | 1653 | 33.4 (30.3-36.7) | 15.7 (13.4-18.3) |
| Dominican Republic | 2016 | 63 | 1252 | 45.7 (41.6-49.9) | 10.1 (8.1-12.5) |
| Ecuador | 2007 | 86 | 2093 | 28.6 (26.3-31) | 7.6 (5.9-9.7) |
| Egypt | 2006 | 87 | 4904 | 23.4 (18.9-28.6) | 8.9 (7.3-10.8) |
| El Salvador | 2023 | 88 | 1819 | 34.5 (29.5-39.7) | 6.8 (5.6-8.1) |
| Fiji | 2016 | 79 | 2813 | 28.3 (25.3-31.4) | 12 (10.7-13.4) |
| French Polynesia | 2015 | 70 | 2723 | 40.5 (37.8-43.3) | 11.5 (10.4-12.7) |
| Ghana | 2012 | 82 | 2352 | 19.5 (17.5-21.6) | 13.2 (11.3-15.3) |
| Grenada | 2008 | 78 | 1370 | 40.7 (37.2-44.4) | 11.7 (9.5-14.3) |
| Guatemala | 2015 | 82 | 3732 | 22.3 (18-27.4) | 6.4 (5.2-7.9) |
| Guyana | 2010 | 76 | 2258 | 36.1 (31.9-40.4) | 14.1 (12.7-15.6) |
| Honduras | 2012 | 79 | 1665 | 30.3 (27.9-32.8) | 5.5 (4.5-6.7) |
| India | 2007 | 83 | 7443 | 22.8 (20.7-25) | 7.8 (7-8.7.0) |
| Indonesia | 2015 | 94 | 10545 | 27.1 (23.8-30.7) | 4.6 (4-5.2.0) |
| Iraq | 2012 | 88 | 1870 | 26.2 (23.2-29.5) | 13.1 (10.6-16) |
| Jamaica | 2017 | 60 | 1487 | 56.7 (52.7-60.6) | 13.2 (11.4-15.3) |
| Jordan | 2007 | 99.8 | 2039 | 39.6 (35.7-43.7) | 18.5 (15.8-21.5) |
| Kenya | 2003 | 84 | 3052 | 37.8 (35.2-40.4) | 14.9 (12.8-17.4) |
| Kiribati | 2011 | 85 | 1521 | 15.1 (13.2-17.3) | 9.2 (7.6-11.0) |
| Kuwait | 2015 | 78 | 2914 | 65.2 (60.2-69.9) | 20.2 (17-23.7) |
| Lao People's Democratic Republic | 2015 | 70 | 3561 | 20.9 (17.3-25) | 4.9 (4.1-5.9) |
| Lebanon | 2017 | 82 | 4817 | 44.6 (41.4-47.9) | 13.4 (12-14.9) |
| Liberia | 2017 | 71 | 1069 | 20.5 (17.1-24.3) | 17 (14.6-19.6) |
| Malaysia | 2012 | 89 | 24912 | 47.4 (45.9-48.9) | 5.3 (4.9-5.7) |
| Mauritius | 2017 | 84 | 2779 | 40.5 (37.6-43.4) | 9.0 (7.7-10.5) |
| Mongolia | 2013 | 88 | 5062 | 44.3 (41-47.6) | 5.4 (4.7-6.3) |
| Morocco | 2016 | 91 | 5397 | 31.0 (27.2-35) | 15.8 (14.4-17.3) |
| Mozambique | 2015 | 80 | 1242 | 38.6 (34.2-43.2) | 9.3 (6.9-12.3) |
| Myanmar | 2016 | 86 | 2698 | 15.9 (13.7-18.3) | 3.55 (2.9-4.2) |
| Namibia | 2013 | 89 | 3168 | 36.2 (33.3-39.1) | 14.1 (12.3-16) |
| Nepal | 2015 | 69 | 6042 | 10.6 (8.5-13.1) | 4.3 (3.4-5.2) |
| Occupied Palestinian territory | 2011 | 96 | 2042 | 25.9 (22.8-29.2) | 13.4 (11.5-15.5) |
| Paraguay | 2017 | 87 | 2803 | 34.7 (30.1-39.7) | 9.1 (7.6-10.7) |
| Peru | 2010 | 85 | 2819 | 28.8 (25.6-32.1) | 8.8 (8.1-9.7) |
| Philippines | 2015 | 79 | 8164 | 31.6 (28.2-35.3) | 10.7 (9.7-11.8) |
| Qatar | 2011 | 87 | 1594 | 47.8 (44.2-51.4) | 17.7 (15.2-20.4) |
| Saint Lucia | 2007 | 82 | 1233 | 53.8 (50-57.7) | 11.1 (9.5-12.9) |
| Saint Vincent and the Grenadines | 2007 | 84 | 1190 | 39.1 (35.6-42.7) | 13.8 (11.6-16.4) |
| Samoa | 2011 | 79 | 1893 | 37.7 (34.4-41.1) | 27.2 (24-30.6) |
| Seychelles | 2015 | 82 | 2328 | 51.1 (48.1-54.1) | 11 (9.7-12.5) |
| Solomon Islands | 2011 | 85 | 1222 | 25.4 (21.3-30) | 12.5 (10.5-14.8) |
| Sri Lanka | 2016 | 89 | 3138 | 37.3 (33.4-41.4) | 4.6 (3.6-5.9) |
| Suriname | 2016 | 83 | 1845 | 43.7 (40.7-46.8) | 12.3 (10.7-14.1) |
| Syrian Arab Republic | 2010 | 97 | 2964 | 25.4 (20.7-30.7) | 14.5 (12.2-17.2) |
| Thailand | 2015 | 89 | 5415 | 56.4 (51.9-60.8) | 8.5 (7.1-10.2) |
| The former Yugoslav Republic of Macedonia | 2007 | 93 | 1938 | 53.1 (47.3-58.9) | 6.4 (5.2-7.7) |
| Timor-Leste | 2015 | 79 | 2866 | 14.8 (12.9-16.9) | 11.3 (9.8-13.1) |
| Tokelau | 2014 | 71 | 120 | 48.0 (40.8-55.2) | 11.3 (7.2-17.3) |
| Tonga | 2017 | 90 | 3023 | 21.1 (19.2-23) | 14.4 (13.0-16.0) |
| Trinidad and Tobago | 2017 | 89 | 3494 | 48.6 (45.4-51.8) | 13.7 (12.2-15.3) |
| Tunisia | 2008 | 83 | 2678 | 24.5 (21.7-27.6) | 19.8 (17.7-22.0) |
| Uganda | 2003 | 69 | 2875 | 26.0 (23.3-29.0) | 11 (9.5-12.8) |
| United Arab Emirates | 2016 | 80 | 5198 | 59.1 (55.5-62.6) | 15.9 (14.4-17.6) |
| United Republic of Tanzania | 2014 | 87 | 3358 | 20.0 (17.4-22.9) | 5.8 (4.7-7.0) |
| Uruguay | 2012 | 77 | 3317 | 59.2 (56.7-61.7) | 5.9 (5.0-6.9) |
| Vanuatu | 2016 | 57 | 1957 | 20.7 (17.7-24.2) | 6.6 (5.4-8.2) |
| Venezuela | 2003 | 85 | 2029 | 28.1 (24.8-31.6) | 3.5 (2.8-4.2) |
| Wallis and Futuna | 2015 | 82 | 929 | 37.0 (33.4-40.8) | 15.3 (13.2-17.7) |
| Yemen | 2014 | 75 | 2104 | 21.4 (16.5-27.4) | 14.7 (12.3-17.3) |
| Zambia | 2004 | 84 | 1669 | 31.0 (28.3-33.8) | 24.4 (22.2-26.7) |

^1^ Response rate was calculated as school response rate multiplied by student response rate.

^2^ Estimates are sex- and age-adjusted.

^3^ ≥3 h of sedentary behavior per day.

^4^ Anxiety-induced sleep disturbance was defined as answering ‘most of the time’ or ‘always’ to the ^5^ question ‘During the past 12 months, how often have you been so worried.

^6^ about something that you could not sleep at night?’

**Table A1.** The prevalence of sedentary behavior (<3h/day and ≥3h/day) at different ages (by overall and sex)

| Age | Overall | | Boy | | Girl | |
| --- | --- | --- | --- | --- | --- | --- |
|  | <3 h/day | ≥3h/day | <3 h/day | ≥3h/day | <3 h/day | ≥3h/day |
|  | %, 95%CI | %, 95%CI | %, 95%CI | %, 95%CI | %, 95%CI | %, 95%CI |
| 11 | 78.3 (75.1-81.3) | 21.7 (18.7-24.9) | 78.8 (74.5-82.5) | 21.2 (17.5-25.5) | 78.0 (73.8-81.6) | 22.0 (18.4-26.2) |
| 12 | 79.0 (77.5-80.4) | 21.0 (19.6-22.5) | 78.9 (77.1-80.7) | 21.1 (19.3-22.9) | 79.0 (77.0-80.9) | 21.0 (19.1-23.0) |
| 13 | 75.4 (73.7-77.0) | 24.6 (23.0-26.3) | 75.5 (73.1-77.8) | 24.5 (22.2-26.9) | 75.2 (73.3-77.1) | 24.8 (22.9-26.7) |
| 14 | 72.1 (70.7-73.5) | 27.9 (26.5-29.3) | 72.7 (70.9-74.4) | 27.3 (25.6-29.1) | 71.5 (69.9-73.1) | 28.5 (26.9-30.1) |
| 15 | 69.5 (67.8-71.1) | 30.5 (28.9-32.2) | 70.6 (68.6-72.5) | 29.4 (27.5-31.4) | 68.3 (66.3-70.2) | 31.7 (29.8-33.7) |
| 16 | 63.9 (61.8-65.9) | 36.1 (34.1-38.2) | 65.9 (63.9-67.8) | 34.1 (32.2-36.1) | 61.5 (58.8-64.2) | 38.5 (35.8-41.2) |
| 17 | 61.8 (58.9-64.7) | 38.2 (35.3-41.1) | 64.1 (61.6-66.5) | 35.9 (33.5-38.4) | 59.5 (55.2-63.7) | 40.5 (36.3-44.8) |

%, the prevalence of sedentary behavior; CI, confidence interval.
